# Identification of histone malonylation in the human fetal brain and implications for diabetes‐induced neural tube defects

**DOI:** 10.1002/mgg3.1403

**Published:** 2020-07-15

**Authors:** Qin Zhang, Tanxi Cai, Zonghui Xiao, Dan Li, Chunlei Wan, Xiaodai Cui, Baoling Bai

**Affiliations:** ^1^ Beijing Municipal Key Laboratory of Child Development and Nutriomics Capital Institute of Pediatrics Beijing China; ^2^ Laboratory of Protein and Peptide Pharmaceuticals & Laboratory of Proteomics Institute of Biophysics Chinese Academy of Sciences Beijing China; ^3^ Weifang Medical University Weifang China

**Keywords:** diabetes, histone lysine malonylation, mass spectrometry, neural tube defects

## Abstract

**Background:**

Neural tube defects (NTDs) are severe congenital malformations. Diabetes during pregnancy is a risk factor for NTDs, but its mechanism remains elusive. Emerging evidence suggests that protein malonylation is involved in diabetes. Here, we report the correlation between histone lysine malonylation in diabetes‐induced NTDs.

**Methods:**

Nano‐HPLC/MS/MS was used to screen the histone malonylation profile in human embryonic brain tissue. Then, the histone malonylation level was compared between the brains of normal control mice and mice with diabetes‐induced NTDs. Finally, the histone malonylation level was compared under high glucose exposure in an E9 neuroepithelial cell line (NE4C).

**Results:**

A total of 30 histone malonylation sites were identified in human embryonic brain tissue, including 18 novel sites. Furthermore, we found an increased histone malonylation level in brain tissues from mice with diabetes‐induced NTDs. Finally, both the histone malonylation modified sites and the modified levels were proved to be increased in the NE4C treated with high glucose.

**Conclusion:**

Our results present a comprehensive map of histone malonylation in the human fetal brain. Furthermore, we provide experimental evidence supporting a relationship between histone malonylation and NTDs caused by high glucose‐induced diabetes. These findings offer new insights into the pathological role of histone modifications in human NTDs.

## Graphical abstract

In this manuscript, we reported, for the first time, the presence of histone malonylation in human fetal brain and mouse neural stem cells.

The results reveal that histone malonylation aberrant in diabetes‐induced NTDs.

These results may bring a new insight in the pathological role of histone modifications in human NTDs.

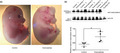

## 1 INTRODUCTION

Human neural tube defects (NTDs) are common, severe, and costly birth defects that arise between the third and fourth weeks of embryogenesis because of partial or complete failure of neural tube closure (Wallingford, Niswander, Shaw, & Finnell, 2013). The incidence of NTDs is approximately 1 in 1,000 births, but in some geographic regions, the incidence is estimated to reach 4–10 in 1,000 (Gu et al., 2007; van der Put, van Straaten, Trijbels, & Blom, 2001). Diabetes during pregnancy is a risk factor for NTDs (Jia et al., 2019; Liu et al., 2019; Salih, Murshid, & Seidahmed, 2014). Even under modern preconception care, diabetic women are three to four times more likely to have a child with these birth defects than healthy women (Correa et al., 2012). It is necessary to understand the mechanisms underlying diabetes‐induced NTDs to implement effective prevention strategies.

Results from recent studies have suggested that abnormal histone malonylation is related to the occurrence of diabetes mellitus type 2 (type 2 diabetes, T2D) (Du et al., 2015). Protein lysine malonylation, a type of posttranslational modification (PTM), which was first identified in Escherichia coli (E. coli) and HeLa cells using a specific anti‐Kmal (anti‐malonyl‐lysine) antibody (Peng et al., 2011), has been shown to play an important role in the development of T2D (Raciti et al., 2014; Slomko, Heo, & Einstein, 2012). Yipeng Du et al performed affinity enrichment coupled with proteomic analysis on liver tissues of both wild‐type (wt) and db/db mice (a typical mouse models of T2D) and identified a total of 573 malonylated lysine sites from 268 proteins. They found that there were more malonylated lysine sites and proteins in db/db than in wt mice. Furthermore, elevation of lysine malonylation of five proteins, including 10‐formyltetrahydrofolate dehydrogenase, glucose‐6‐phosphate isomerase (G6PI), fructose‐1,6‐bisphosphatase 1 (FBP1), L‐lactate dehydrogenase A chain (LDHA), and glutathione S‐transferases, were verified in db/db mice. Among them, G6PI, FBP1, and LDHA are important enzymes in glucose metabolism and support a potential role of protein lysine malonylation in T2D (Du et al., 2015). Histone lysine malonylation was also observed in T2D. In the brain of a high‐fat‐diet‐induced obese mouse model (DIO), 170 histone markers were identified. Among them, H3K23, H3K56, H3K79, H2BK108, H2BK116, and H2BK120 were modified by malonylation, providing a resource for studying the epigenetic functions of histone modifications caused by obesity and T2D (Nie et al., 2017).

In the present study, we aimed to determine whether histone malonylation is involved in diabetes‐induced NTDs. Using Nano‐HPLC/MS/MS analysis, we observed the number of histone lysine malonylation sites in human brain tissues. Then, we compared the histone malonylation levels between mice with high glucose‐induced NTDs and normal control mice. We found that the malonylation level was increased in mice with high glucose‐induced NTDs. Furthermore, we compared the histone malonylation levels between high glucose‐treated mouse neural stem cells (NE4C) and normal cells. We found an abundance of histone lysine malonylation sites in NE4C cells. After treatment with 25 mM glucose, the histone malonylation level was increased compared with normal cells (5 mM glucose) according to parallel reaction monitoring (PRM) analysis and western blotting. Both the in vivo and in vitro experimental results suggested possible new connections between increased histone lysine malonylation and diabetes‐induced NTDs.

## 2 METHODS AND MATERIALS

### 2.1 Ethical compliance

This investigation was approved by the Medical Ethics Committee of the Capital Institute of Pediatrics. Written informed consent was obtained from all mothers who participated in this study.

### 2.2 Human subjects

The NTDs and normal control sample tissues were obtained from patients in the Lüliang area of Shanxi Province in northern China. Medically aborted fetuses with NTDs that had been diagnosed with encephalocele by B‐mode ultrasound in the early stages of pregnancy were subjected; the sex, gestational age, and general development were also recorded in detail. The pathological diagnosis of NTDs was performed by experienced pathologists in accordance with the *International Classification of Disease, Tenth Revision*, codes Q00.0, Q05.9, and Q01.9. Control fetuses that had been aborted for non‐medical reasons were obtained from patients in the same region (Wang et al., 2010; Zhang et al., 2008). The details of these six samples are shown in Table 1.

**Table 1:** Clinical information of the six individual fetuses

No.	Source of brain tissue	Maternal Glucose level (mM)	Gender	Gestational age (weeks)
1	Normal control	4.33	F	19
2	Normal control	4.28	F	20
3	Normal control	3.91	M	20
4	Encephalocele	7.09	F	17
5	Encephalocele	11.9	M	31
6	Encephalocele	8.24	M	18

### 2.3 Animal experiments

As described in our previous paper (Bai et al., 2018), diabetes was induced in 7‐ to 9‐week‐old virgin female FVB mice by two intraperitoneal injections of 100 mg/kg body weight streptozotocin (STZ; CAS18883‐66‐4, Sigma) in 100 mM sodium citrate buffer at pH 4.5 performed at an interval of 1 week. After fasting for 12 hr, 11 dams were confirmed as having diabetes when the blood glucose level exceeded 14 mM (250 mg/dl) after STZ treatment. Ten female mice were treated with the same volume of vehicle (100 mM sodium citrate buffer) and included in the normal control group. The dams were then mated with 8‐ to 11‐week‐old male FVB mice. The day that a vaginal plug was detected was considered day 0.5 of gestation. On gestational day 14.5, the dams were euthanized by CO_2_ for subsequent experiments.

### 2.4 Cell culture and glucose treatment

As described in our previous paper (Bai et al., 2018), NE4C cells (Schlett & Madarasz, 1997), purchased from the Stem Cell Bank, Chinese Academy of Science, were cultured on plates coated with 15 mg/ml poly L‐lysine 2 hr before passage in Eagle's MEM (Gibco) supplemented with 10% fetal bovine serum (FBS) (Cat No: 10099; lot number: 1581729; Thermo‐Fisher), 1% GlutaMAX (Invitrogen), and 1% non‐essential amino acids (Invitrogen) at 37°C in 5% CO2. To stimulate high glucose exposure, cells were incubated in serum‐free medium for 24 hr, and then 5 mM (normal control) or 25 mM (high) glucose (G7020, Sigma) were added in each group and both with FBS reintroduced (Wellen et al., 2009), cells were further cultured 24 hr for following experiments.

### 2.5 Histone extraction from NE4C cells

Core histone proteins were extracted from NE4C cells using an acid extraction protocol (Hake, Shechter, Dormann, & Allis, 2007). The samples were first homogenized in lysis buffer (10 ml solution containing 10 mM Tris‐Cl pH 8.0, 1 mM KCl, 1.5 mM MgCl_2_, and 1 mM dithiothreitol [DTT]) and chilled on ice. Protease and phosphatase inhibitors were added immediately before cell lysis, and nuclei were isolated by centrifugation (1,500 *g* for 10 min). For the preparation of histones, nuclei were incubated with four volumes of 0.2 N sulfuric acid overnight at 4°C. The supernatant was precipitated with 33% trichloroacetic acid followed by centrifugation (12,000 *g* for 20 min). The obtained pellet was washed with cold acetone and subsequently dissolved in distilled water. The samples were stored at −80°C before analysis (as described in our previous paper) (Zhang et al., 2013).

### 2.6 In‐solution tryptic digestion

As described in our previous paper (Zhang et al., 2013), 40 μg of core histone protein mixture was extracted from NE4C cells and digested as follows. Disulfide bonds were reduced with 10 mM (final concentration) DTT for 60 min at 37°C. Then, alkylation was carried out by adding 40 mM (final concentration) iodoacetamide for 60 min at room temperature in the dark. The alkylation reaction was quenched by treatment with 40 mM DTT for 15 min. After diluting urea to less than 1 M with 25 mM NH4HCO3, sequence‐grade trypsin was added at a ratio of 1:40 (enzyme:total protein), and proteins were then digested at 37°C for 4 hr. The tryptic digestion was quenched by adding 1.0% trifluoroacetic acid, and the solution was then centrifuged at 13,000 *g* for 10 min to remove insoluble material. The supernatant was collected for subsequent experiments.

### 2.7 Identification and quantification of histone lysine malonylation by Nano‐HPLC/MS/MS

Nano‐HPLC/MS/MS analyses were performed on a Q Exactive HF mass spectrometer (Thermo Scientific, Bremen, Germany) equipped with an UltiMate 3000 RSLCnano System (Dionex, Germering, Germany). Full‐scan MS spectra in the m/z range of 350–2,000 were acquired using an Orbitrap. Twenty of the most intense ions were isolated for MS/MS analysis. The raw data were processed using Proteome Discoverer (version 2.1.0.81, Thermo Fisher Scientific) by searching a database of human histones (www.uniprot.org, accessed October 2015) (Zhang et al., 2018). Peptides were generated from a semi‐tryptic digestion with up to four missed cleavages, using carbamidomethylation of cysteines as a fixed modification and oxidation of methionines as a variable modification. Target histone lysine malonylation was searched at 86.00039 Da (Xie et al., 2012).The precursor mass tolerance was 20 ppm, and the product ions were searched at a tolerance of 0.05 Da. Peptide spectral matches were validated using a percolator based on q‐values at a 1% false discovery rate (FDR). Modified peptides that passed the FDR were exported to a text file and processed by PRM. The area of the peaks was used to represent the number of modifications (as shown in our previous paper) (Zhang et al., 2018).

### 2.8 Parallel reaction monitoring (PRM)

Raw data were searched against the corresponding histone database. Modifications including lysine malonylation, acetylation, and mono‐, di‐, and trimethylation were searched. The mass inclusion list included the mass, charge, polarity, and time from the start and end. The full scan method was used as described above. The PRM method employed an Orbitrap resolution of 30,000 (at m/z 350) and a target automatic gain control value of 2 × 10^5^. The precursor ions of each peptide were duplexed using ±0.8 m/z unit windows. Each sample was analyzed in triplicate (as shown in our previous paper) (Zhang et al., 2018).

### 2.9 PRM data analysis

Histone PTM quantification was manually processed within the Xcalibur Qual Browser (version 4.0.27.19; Thermo Fisher Scientific) using Skyline (version 3.5.0.9319; AB Sciex). In the Xcalibur Qual Browser, determination of the area under the curve of selected fragment ions was based on the presence of product ion signals within ±2.5 min of the expected retention time, with a mass error within ±5 ppm. In Skyline,.raw files were used as input to generate and extract the modified peptide normalized area at a 0.05 m/z ion match tolerance for each PRM spectrum (as shown in our previous paper) (Zhang et al., 2018).

### 2.10 Western blotting

Total lysate histone samples were resolved by 15% SDS‐PAGE and subjected to western blot assays with a rabbit polyclonal anti‐lysine malonylation antibody (14942, Cell Signaling Technology, 1:2,000), followed by incubation with an anti‐rabbit horseradish peroxidase‐conjugated antibody (SC‐2048, Zhongshan Jinqiao, 1:5,000) and detection with a West Pico ECL kit (Thermo Scientific). Purified Histones H3 were purchased from New England Biolabs (M2503S).

### 2.11 Statistical analysis

Statistical parameters for each experiment are reported in the corresponding figures.

## 3 RESULTS

### 3.1 Mass spectrometry mapping of histone lysine malonylation in human fetal brains

**Figure 1:**
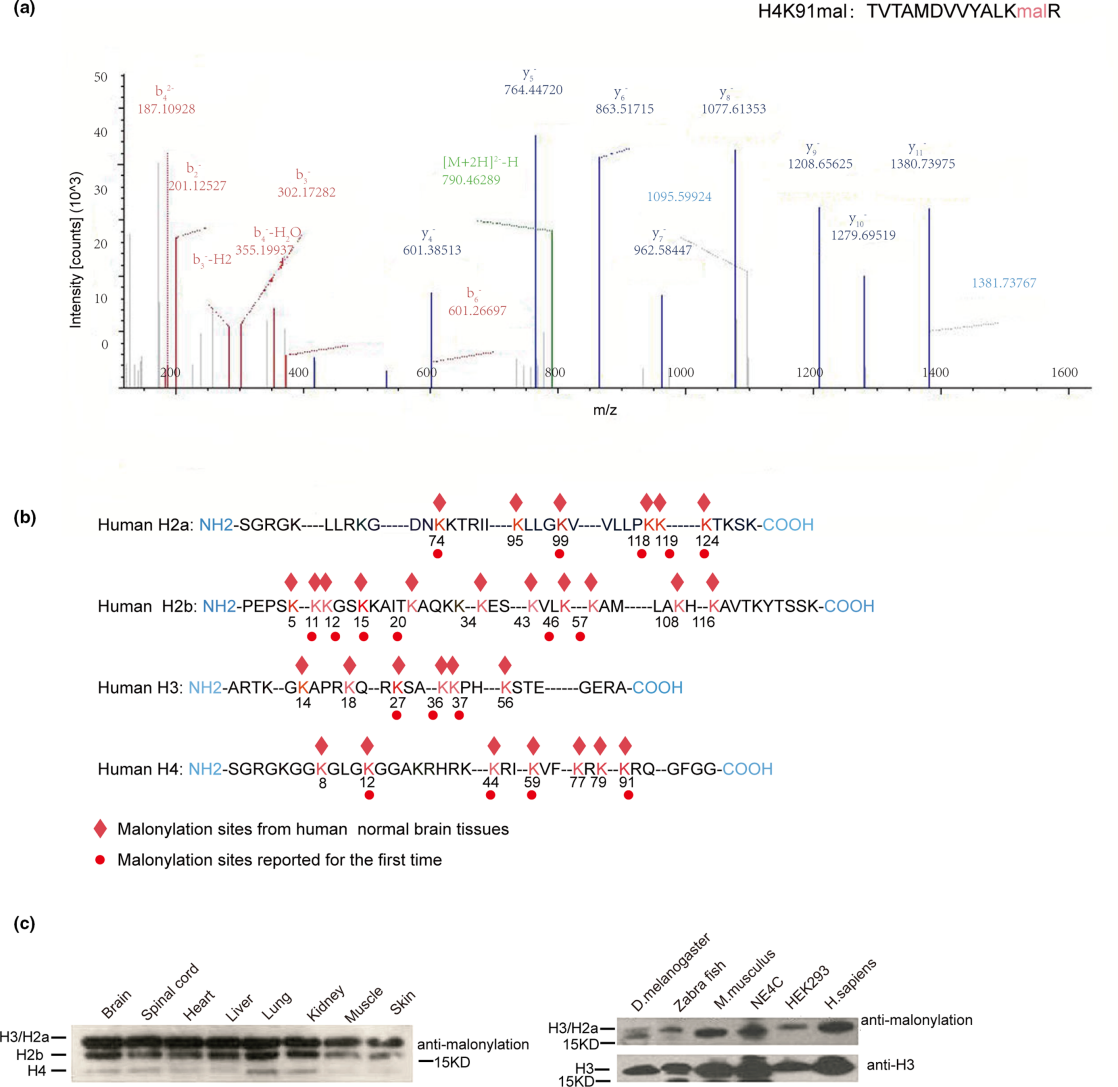
Profile of histone malonylation in normal human fetal brain tissues. (a) A typical HPLC‐MS/MS spectra of a tryptic peptide “TVTAMDVVYALKmalR” harboring H4K91 malonylation, derived from human fetal brain. The x‐ and y‐axes represent m/z and relative ion intensity, respectively. A series of b‐ and y‐type malonylation fragment ions are evident which not only provide reliable sequence information but also indicate an unambiguous shift for malonylation. The x‐ and y‐axes represent m/z and relative ion intensity, respectively. (b) Schematics to show residues details of core histones (H3, H4, H2a, and H2b) malonylated sites. Red symbols indicate malonylation modified sites in normal human fetal tissues. (c) (Left panel) Western blotting analysis for the detection of lysine malonylation in samples from a variety of human fetal tissues, including brain, spinal cord, heart, liver, lung, kidney, muscle, and skin. (Right panel) Presence of lysine malonylation in different species, including D. melanogaster, Zebra fish, M. musculus, NE4C, HEK293, and brain tissues

To systematically analyze histone lysine malonylation sites in humans, histones from six fetal brains were isolated by acid extraction, tryptically digested and subjected to nano‐HPLC‐MS/MS analysis. A representative LC‐MS/MS spectrum of the identified malonylation‐modified peptide “TVTAMDVVYALK_mal_R (H4K91mal)” is shown in Figure 1a. A series of b‐ and y‐type malonylation fragment ions provided reliable sequence information and indicated an unambiguous +86.00039 Da shift for lysine. In all, 30 histone lysine malonylation sites were identified in core histones (H2a, H2b, H3, and H4) (Figure 1b and Table 2). Among these sites, 12 histone lysine malonylation sites, including H3K14, H3K18, H3K56, H4K8, H4K77, H4K79, H2aK95, H2bK5, H2bK34, H2bK43, H2bK108, and H2bK120, had been detected in previous reports (Nie et al., 2017; Sabari, Zhang, Allis, & Zhao, 2017). However, 18 histone lysine malonylation sites, including H3K27, H3K36, H3K37, H4K12, H4K44, H4K59, H4K91, H2aK74, H2aK99, H2aK118, H2aK119, H2aK124, H2bK11, H2bK12, H2bK15, H2bK20, H2bK46, and H2bK57, were reported for the first time (the novel modified sites marked with red dot in Figure 1b). Taken together, these data identified a novel‐specific histone lysine malonylation map in the human brain. Our results suggested that malonylation is a relatively common histone marker. In addition, most of the identified histone malonylation sites have been shown to be subjected to other types of modifications, including H3K27, H3K36, and H3K56, which are sites for which modification is important for chromatin structure and function. We compared the histone lysine malonylation sites between NTDs with high maternal glucose level and controls with normal maternal glucose level (for six human brain sample details, see Table 1), but we did not find any histone lysine malonylation site only in normal group or NTDs group (for the histone malonylation distribution of samples, see Table S1).

**Table 2:** Histone peptides including lysine malonylation identified using MS in human fetal brain tissues

Protein Name	Modification Site	Peptide sequence and modification	MH+[Da]^a^	Confidence^b^
H2a	K74	ELAGNAARDNKmalKTR	909.43536	High
	K95	IRNDEELNKmalLLGKVTIAQGGVLPNIQAVLLPK	3554.08811	High
		NDEELNKmalLLGKVTIAQGGVLPNIQAVLLPK	1,090.95288	High
		HLQLAIRNDEELNKmalLLGK	548.31000	High
	K99	IRNDEELNKLLGKmalVTIAQGGVLPNIQAVLLPK	1185.36755	High
		NDEELNKLLGKmalVTIAQGGVLPNIQAVLLPK	1090.95483	High
	K118	VTIAQGGVLPNIQAVLLPKmalKTESQK	690.90485	High
	K119	VTIAQGGVLPNIQAVLLPKKmalTESQK	920.87042	High
	K124	PNIQAVLLPKKTESQKmalTKSK	789.43445	High
H2b	K5	PEPSKmalSAPAPKKGSK	546.30475	High
	K11	APAPKmalKGSKKAITK	1636.92302	High
	K12	KKmalGSKKAITK	674.87665	High
	K15	KKGSKmalKAITK	1348.74443	High
	K20	KAITKmalAQK	1254.54221	High
	K34	SRKmalESYSIYVYKVLKQVHPDTGISSK	3154.68984	High
		KRSRKmalESYSIYVYK	1921.02665	High
	K43	SRKESYSIYVYKmalVLKQVHPDTGISSK	3168.70669	High
		KESYSIYVYKmalVLKQVHPDTGISSK	2939.49973	High
		SYSIYVYKmalVLKQVHPDTGISSK	2626.33609	High
		KmalVLKQVHPDTGISSK	1750.94390	High
	K46	VLKmalQVHPDTGISSKAMGIMNSFVNDIFER	3347.69785	High
		SYSIYVYKVLKmalQVHPDTGISSK	2626.33609	High
		KVLKmalQVHPDTGISSK	1750.94390	High
	K57	VLKQVHPDTGISSKmalAMGIMNSFVNDIFER	3347.69785	High
		QVHPDTGISSKmalAMGIMNSFVNDIFER	2979.43338	High
		PDTGISSKmalAMGIMNSFVNDIFER	872.41479	High
	K108	EIQTAVRLLLPGELAKmalHAVSEGTKAVTKY	3278.87342	High
		REIQTAVRLLLPGELAKmalHAVSEGTKAVTK	3243.85390	High
		NKRSTITSREIQTAVRLLLPGELAKmal	2922.61832	High
		LLLPGELAKmalHAVSEGTKA	1962.05669	High
		LPGELAKmalHAVSEGTK	1622.86760	High
	K116	EIQTAVRLLLPGELAKHAVSEGTKmalAVTKY	3278.87342	High
		LLLPGELAKHAVSEGTKmalA	1962.05669	High
H3	K14	ARKSTGGKmalAPR	628.84601	High
	K18	KSTGGKAPRKmalQLA	1441.79009	High
	K27	KAARKmalSAPATGGVKKPHR	2162.22654	High
		KQLATKAARKmalSAPATGGVK	2143.14621	High
		AARKmalSAPATGGVKKPHR	1932.04006	High
	K36	AARKSAPATGGVKmalKPHRYRPGT	2434.30899	High
		QLATKAARKSAPATGGVKmalKPHR	2401.35088	High
		TKAARKSAPATGGVKmalKPHR	2235.22849	High
		VKmalKPHR	936.49457	High
	K37	KSAPATGGVKKmalPHRYRPGTV	2249.29099	High
		KAARKSAPATGGVKKmalPHR	721.39667	High
		SAPATGGVKKmalPHRYRPGTV	2121.15470	High
	K56	RRYQKmalSTELLIR	1690.93521	High
H4	K8	MSGRGKGGKmalGLGKGGAK	1787.91362	High
		KGGKmalGLGK	872.51459	High
	K12	KGGKGLGKmalGGAKR	1441.79009	High
		MSGRGKGGKGLGKmalGGAK	1787.91362	High
		SGRGKGGKGLGKmalGGAK	1674.89518	High
		KGLGKmalGGAKRHR	1594.89568	High
	K44	GGVKmalRISGLIYEETRGVLKVFLENVIR	3201.85621	High
		VKmalRISGLIYEETRGVLKVFLENVIR	3045.75730	High
		RRGGVKmalRISGLIYEETR	2132.19541	High
		RGGVKmalRISGLIYEETRGV	2118.17960	High
		GVKmalRISGLIYEETRGVLK	2104.18935	High
	K59	RISGLIYEETRGVLKmalVFLENVIRDAVTYTEHAK	3976.19799	High
		ISGLIYEETRGVLKmalVFLENVIRDAVTYTEHAKR	3,948.18066	High
		YEETRGVLKmalVFLENVIRDAVTYTEHAK	3294.75405	High
		ISGLIYEETRGVLKmalVFLENVIR	2662.44193	High
		EETRGVLKmalVFLENVIR	2002.12058	High
	K77	ISGLIYEETRGVLKVFLENVIRDAVTYTEHAKmalR	3948.18281	High
		TYTEHAKmalRKTVTAMDVVYALK	2685.32517	High
		YTEHAKmalRKTVTAMDVVYALK	2584.33353	High
		HAKmalRKTVTAMDVVYALKR	2201.21573	High
	K79	HAKRKmalTVTAMDVVYALKR	734.41681	High
		TYTEHAKRKmalTVTAMDVVYALK	2685.32517	High
	K91	KTVTAMDVVYALKmalRQGRTLYGF	2631.41079	High
		KTVTAMDVVYALKmalRQGRTLYG	2540.41129	High
		TVTAMDVVYALKmalRQGRTLY	2271.16557	High
		TVTAMDVVYALKmalR	1594.85454	High

^a^ Protonated MW of the Primary Sequence.

^b^ Refers to high scoring peptide evaluated with PD software.

To confirm the presence of histone malonylation, western blot assay was performed, and a significant malonylation signal was observed in histones from human embryonic brain tissues (Figure 1c, left panel). A histone lysine malonylation signal was also detected in samples from other human embryonic tissues including the spinal cord, heart, lung, kidney, liver, muscle, and skin. To further determine whether lysine malonylation is present in a broad range of species, we performed western blot assays using a lysine malonylation‐specific antibody on samples from Drosophila melanogaster, zebrafish and Mus musculus, which were compared with Homo sapiens. Our results showed that the lysine malonylation signal could be detected in all of these samples (Figure 1c, right panel). These findings suggest that histone lysine malonylation is an evolutionarily conserved modification across a wide range of species and may have a potential biological function.

### 3.2 The histone malonylation expression level is increased in mice with diabetes‐induced NTDs

**Figure 2:**
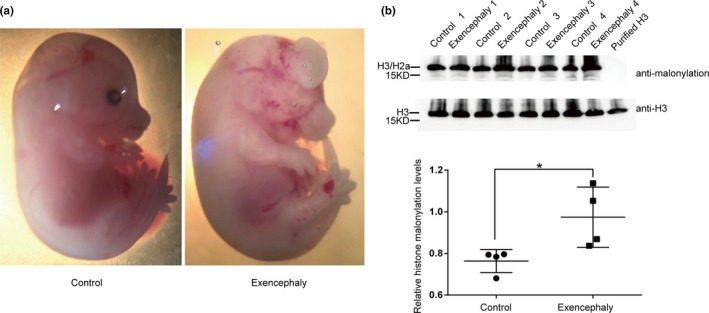
Increased histone malonylation in mouse maternal diabetes‐induced neural tube defects. (a) Maternal diabetes induced E14.5 mouse embryos exhibit exencephaly (right panel), while control female FVB mice produce normal phenotype (left panel). (b) Western blotting analysis for the detection of histone malonylation in brain tissues from control and mouse maternal diabetes induced exencephaly, respectively. The bands (H3/H2b) were the targets to quantification. The relative level of histone malonylation was normalized to H3 level. Data represent mean ± SEM. The *p* values were calculated with unpaired *t* test. *p* value of exencephaly versus control tissues is .03 (*p < .05). In the control group, the relative histone malonylation levels were 0.763 ± 0.028 *N* = 4. In the exencephaly group, the relative histone malonylation levels were 0.974 ± 0.072 *N* = 4

To examine whether histone malonylation in brain tissue is associated with NTDs induced by diabetes, we constructed a mouse model of diabetes‐induced NTDs. STZ was used to induce diabetes in FVB female mice, which were mated with normal male mice. Embryos were extracted at E14.5. 80.5% of mouse embryos are normal. 2.6% of mouse embryos are other abnormal phenotypes. Spina bifida was found in 2.6% of mouse embryos. Exencephaly was found in 14.3% of mouse embryos. Mouse embryos with exencephaly show appearance of failed cranial neural tube closure with a region of everted, open neural folds involving forebrain, midbrain, or hindbrain. And the brain tissues of mice with exencephaly were used to extract histones to test the histone malonylation level (Figure 2a).

The histone malonylation levels of brain tissues from control embryos and those embryos with NTDs were detected by western blots. A marked elevation of histone malonylation was observed in mice with NTDs relative to their control embryos littermates (Figure 2b). Equal loading was verified by H3 western blots. These results remind us that altered histone malonylation is potentially associated with pathogenesis of diabetes‐induced NTDs.

### 3.3 Increase in histone lysine malonylation levels in high glucose exposure in mouse neural stem cells

**Figure 3:**
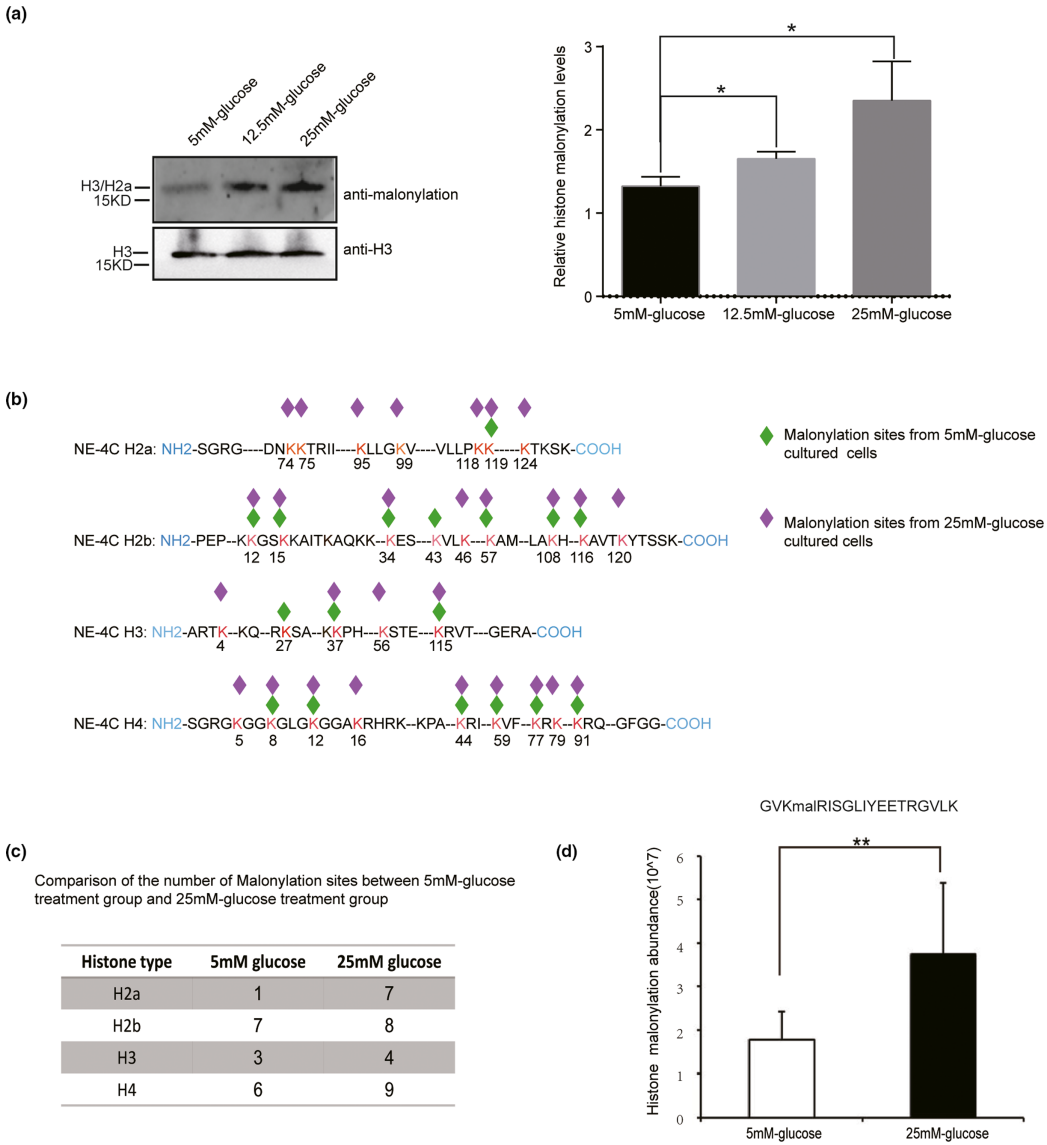
Elevated histone malonylation in NE4C exposed to high glucose. (a) Western blotting analysis of lysine malonylation in NE4C cells treated with increasing doses of glucose. The data presented were derived from three independent experiments and were reported as standard error of the mean (SEM). The relative level of histone malonylation were normalized to H3 level. In the 5 mM glucose treatment group, the relative histone malonylation levels were 1.323 ± 0.065 *N* = 3. In the 12.5 mM glucose treatment group, the relative histone malonylation levels were 1.653 ± 0.050 *N* = 3. In the 25 mM glucose treatment group, the relative histone malonylation levels were 2.350 ± 0.273 *N* = 3. The p values were calculated with unpaired *t* test. *p* value of 5 mM glucose treatment group versus 12.5 mM glucose treatment group is .016 (*p < .05); *p* value of 5 mM glucose treatment group versus 25 mM glucose treatment group is .022 (*p < .05). (b) A summary of malonylated lysine sites of core histones (H2a, H2b, H3, and H4) in NE4C cells exposed to normal glucose (5 mM‐G) and high glucose (25 mM‐G) by HPLC‐MS/MS analysis. The detected malonylation sites from high glucose or normal glucose are shown in purple or green diamond shape, respectively. The number underneath each red lysine residue (K) represents the position of the particular lysine residue within each respective histone. (c) Comparison of the number of malonylation sites between normal glucose (5 mM glucose) and high glucose (25 mM glucose) in histone H3, H4, H2a, and H2b. (d) Relative histone H4K44 (GVKmalRISGLIYEETRGVLK) malonylated levels in 5 mM, 25 mM glucose‐treated NE4C cells by Skyline quantitative analysis upon HPLC‐MS/MS results. Data represent mean ± SEM (*n* = 3). **p < .01 versus 5 mM‐G; y‐axis represents histone modification abundance

To further clear the effect of high glucose exposure on histone malonylation, cells from the mouse neural stem cell line NE4C were treated with 5, 12.5, or 25 mM glucose. Western blot assays of extracted histones revealed that histone malonylation levels increased with increasing glucose concentration (Figure 3a), suggesting a direct effect of the glucose dose on histone malonylation. To confirm the effect of glucose on histone malonylation, histones from cells treated with 5 mM and 25 mM glucose were analyzed by Nano‐HPLC/MS/MS. The results showed 17 malonylation sites identified in the 5 mM glucose treatment group and 28 malonylation sites identified in the 25 mM glucose treatment group. Among the malonylation sites, 15 overlapped between the two groups (Figure 3b,c, Table S2).Among these sites, 24 lysine malonylation sites were the same as those detected in human fetal tissue. Nine histone lysine malonylation sites, including H3K56, H4K8, H4K77, H4K79, H2aK95, H2bK34, H2bK43, H2bK108, and H2bK120, had been detected in previous reports (Nie et al., 2017; Sabari et al., 2017). However, 21 histone lysine malonylation sites, including H3K4, H3K27, H3K37, H3K115, H4K5, H4K12, H4K16, H4K44, H4K59, H4K91, H2aK74, H2aK75, H2aK99, H2aK118, H2aK119, H2aK124, H2bK12, H2bK15, H2bK46, H2bK57, and H2bK116, were reported for the first time. These results showed that more histone lysine sites were malonylated in cells treated with 25 mM glucose than in those treated with 5 mM glucose, suggesting that the histone lysine malonylation level increases with increasing glucose concentration.

Furthermore, the level of histone malonylation was also quantified using a mass spectrometry label‐free method. Figure 3d shows that the level of histone malonylation peptide of H4K44 (GVKmalRISGLIYEETRGVLK) was increased in cells treated with 25 mM glucose compared with those treated with 5 mM glucose. The change in H4K44 malonylated levels between 5 mM and 25 mM is specific. These results suggest that histone malonylation levels are regulated by the glucose level in vitro.

## 4 DISCUSSION

At present, the incidence of diabetes mellitus (including type I and T2D) is increasing annually in China. Approximately 10% of women of childbearing age have diabetes mellitus. Hyperglycemia will not only cause complications in pregnant women but will also lead to the occurrence of NTDs. NTDs are a common congenital malformation, the risk of which increases by approximately 2–5 times because of maternal diabetes, which will seriously affect the birth and health of newborns (Greene & Copp, 2014; Kennelly & McAuliffe, 2016; Ornoy, Reece, Pavlinkova, Kappen, & Miller, 2015; Ramya, Shyamasundar, Bay, & Dheen, 2017; Sukanya, Bay, Tay, & Dheen, 2012). According to an epidemiological survey, maternal diabetes is closely related to an abnormal rate of NTDs (including open brain malformation and spina bifida), and the odds ratio is approximately 2 (Biggio, Chapman, Neely, Cliver, & Rouse, 2010; Rasmussen, Chu, Kim, Schmid, & Lau, 2008; Stothard, Tennant, Bell, & Rankin, 2009; Watkins, Scanlon, Mulinare, & Khoury, 1996; Werler, Louik, Shapiro, & Mitchell, 1996). If the glucose level of the mother is effectively controlled to a normal level in early pregnancy, the rate of fetal malformation can be significantly reduced from 10.9% to 1.2% (Warner, Smith, Smolenkova, Pisano, & Greene, 2016).

However, the mechanism of fetal NTDs caused by maternal diabetes is still not very clear. Previous studies showed that elevated oxidative stress, suppression on autophagy, and neuroepithelial cell apoptosis were thought to be direct consequences for maternal diabetes‐induced NTDs (Wang et al., 2017; Wu et al., 2015; Zhao, Cao, & Reece, 2017). Maternal diabetes may participate in NTDs by altering the expression of genes involved in the signaling and metabolic pathways implicated in embryogenesis. These include genes in the Wnt‐planar cell polarity pathway (Wnt‐PCP), and those involved in folate metabolism, oxidative stress, apoptosis, or proliferation (Dheen et al., 2009; Horal, Zhang, Stanton, Virkamaki, & Loeken, 2004; Pavlinkova, Salbaum, & Kappen, 2009; Phelan, Ito, & Loeken, 1997; Piedrahita et al., 1999; Sato et al., 2008). But it is not clear yet how maternal diabetes affects the expression of these genes. In this study, we identified 30 histone lysine malonylation sites (on core histones) in human fetal brain tissues and showed that elevation of histone malonylation was implicated in diabetes‐induced NTDs in mice. The elevation of lysine malonylation may be involved in the formation of NTDs by regulating genes and pathways related to NTDs.

Histone PTMs give rise to a so‐called “histone code” that is believed to control the recruitment of transcription and/or repression factors, thereby regulating the degree of transcription of the genome. This “code” is highly dynamic, and the modified histones should perhaps be considered as nodes in a dynamic network (Olsen, 2012). Histone PTMs can directly modulate the packaging of chromatin by altering the chemical structures of histones or internucleosomal interactions through changes in the net charge, hydrogen bonding, size, or hydrophobicity in substrate PTM residues (Zeng & Zhou, 2002). Misregulation of histone PTM patterning has been intimately linked with a number of diseases, including developmental and neurological disorders as well as various etiologies of cancer (Sabari et al., 2017). Our previous studies have shown that some NTDs are caused by low maternal folate levels through histone methylation modification, and some NTDs are caused by high maternal homocysteine levels through histone H3K79 homocysteinylation (Zhang et al., 2018).

Histone lysine malonylation is a type of histone acylation that mediates physiological functions, including signal‐dependent gene activation, spermatogenesis, tissue injury, and metabolic stress (Xie et al., 2012). Dysregulation of the homeostasis of short‐chain CoAs, such as malonyl‐CoA, contributes to the development of diabetes (An et al., 2004; Bandyopadhyay, Yu, Ofrecio, & Olefsky, 2006; Nolan, Madiraju, Delghingaro‐Augusto, Peyot, & Prentki, 2006). Elevated malonyl‐CoA levels have been found in T2D patients (Bandyopadhyay et al., 2006) and pre‐diabetic rats (Zhao et al., 2009). By examining high glucose‐exposed neural stem cells in vitro and diabetes‐induced mouse NTDs in vivo, we found a significant elevation of lysine malonylation. Our results provide a possible link between diabetes development and epigenetic regulation through novel histone malonylation sites.

It should be noted that there is no change of malonylated sites in human fetal brain tissue while there are more histone lysine sites were malonylated in cells treated with 25 mM glucose than in those treated with 5 mM glucose. In the NE4C cell line, the significant histone lysine malonylation difference observed under high glucose exposure (treated glucose level were five times higher than normal control). But in the human samples, the difference of maternal glucose level was not so large. We speculate that this is the reason for the difference in results in human and NE4C cell line.

## 5 CONCLUSION

In summary, this study presents a comprehensive map of histone malonylation in the human fetal brain and provides experimental evidence supporting a relationship between histone malonylation and NTDs. Combined with our previous work showing that aberrant histone methylation, histone acetylation, and histone homocysteinylation are involved in NTDs (Li et al., 2019; Zhang et al., 2013), these results confirm that aberrant histone modification during early pregnancy is associated with the occurrence of NTDs.

## CONFLICT OF INTEREST

All authors declare that no competing interests exist.

## AUTHORS’ CONTRIBUTIONS

B.L.B and X.D.C initiated and conceived the study; Q.Z, Z.H.X., and T.X.C designed and performed the experiments; B.L.B and Q.Z. wrote the manuscript; D.L.C.L.W. and D.L. did the bioinformatics and biostatistics analysis under the guidance of B.L.B and X.D.C; T.X.C and Q.Z. contributed to mass spectrometric analysis; D.L. and Z.H.X. contributed to animal models and animal analysis. All authors have read, edited, and approved the final version of the manuscript.

## Supporting information

Table S1‐S2

## Data Availability

All data reported here are available upon request.
