# Supplementary material for: Identification of histone malonylation in the human fetal brain and implications for diabetes‐induced neural tube defects
Source: Mol Genet Genomic Med. 2020 Jul 15;8(9):e1403. doi: 10.1002/mgg3.1403 (PMC7507309; doi:10.1002/mgg3.1403)
Supplement: Supplementary file 1 — Table S1‐S2 [file MGG3-8-e1403-s001.docx]

Supplementary Table 1. The histone malonylation distribution of 6 human brain samples.Samples 1 to 3 were the normal control group, and samples 4 to 6 were the maternal hyperglycemia related NTDs group.

| Protein Name | Modification Site | Normal | | | NTDs | | |
| --- | --- | --- | --- | --- | --- | --- | --- |
|  |  | 1 | 2 | 3 | 4 | 5 | 6 |
| H2A | K74 | ● | ● | ● | ○ | ● | ● |
|  | K95 | ○ | ● | ○ | ● | ○ | ● |
|  | K99 | ● | ● | ○ | ● | ○ | ● |
|  | K118 | ○ | ● | ● | ○ | ● | ○ |
|  | K119 | ● | ● | ● | ● | ● | ● |
|  | K124 | ● | ● | ○ | ○ | ○ | ● |
| H2B | K5 | ○ | ● | ● | ○ | ○ | ● |
|  | K11 | ● | ○ | ● | ○ | ● | ● |
|  | K12 | ● | ● | ○ | ● | ● | ○ |
|  | K15 | ○ | ● | ● | ○ | ● | ○ |
|  | K20 | ○ | ○ | ● | ○ | ● | ● |
|  | K34 | ● | ○ | ● | ○ | ● | ○ |
|  | K43 | ● | ○ | ● | ● | ● | ○ |
|  | K46 | ● | ○ | ● | ● | ● | ○ |
|  | K57 | ● | ● | ○ | ● | ○ | ● |
|  | K108 | ● | ○ | ● | ● | ● | ● |
|  | K116 | ● | ○ | ● | ● | ● | ○ |
| H3 | K14 | ● | ○ | ● | ● | ● | ● |
|  | K18 | ● | ● | ○ | ○ | ● | ○ |
|  | K27 | ● | ● | ○ | ○ | ● | ● |
|  | K36 | ● | ○ | ● | ○ | ● | ○ |
|  | K37 | ○ | ● | ● | ● | ● | ○ |
|  | K56 | ○ | ● | ● | ● | ● | ○ |
| H4 | K8 | ○ | ● | ○ | ○ | ○ | ○ |
|  | K12 | ● | ● | ● | ● | ● | ● |
|  | K44 | ○ | ● | ○ | ○ | ● | ○ |
|  | K59 | ○ | ● | ○ | ● | ● | ○ |
|  | K77 | ● | ● | ○ | ● | ● | ● |
|  | K79 | ○ | ● | ● | ● | ● | ● |
|  | K91 | ● | ● | ○ | ○ | ● | ● |

● the modification in this peptide was detected;

○ the modification in this peptide was not detected.

Supplementary Table 2. Histone peptides including lysine malonylation identified using MS in NE4C.

| **Protein Name** | **Modification Site** | **25mM-glucose** | | **5mM-glucose** | |
| --- | --- | --- | --- | --- | --- |
|  |  | **Peptide sequence and modification** | **MH+[Da]*** | **Peptide sequence and modification** | **MH+[Da]*** |
| H2a | K74 | VGAGAPVYLAAVLEYLTAEILELAGNAARDNKmalKTR | 752.41644 | _ | _ |
|  | K75 | PVYLAAVLEYLTAEILELAGNAARDNKKmalTR | 1139.6283 | _ | _ |
|  | K95 | RHLQLAIRNDEELNKmalLLGKVTIAQGGVLPNIQAVLLPK | 1107.90588 | _ | _ |
|  |  | DEELNKmalLLGKVTIAQGGVLPNIQAVLLPK | 1057.59692 | _ | _ |
|  | K99 | LGKmalVTIAQGGVLPNIQAVLLPK | _ | _ | _ |
|  |  | LQLAIRNDEELNKLLGKmalVTIAQGGVLPNIQAVLLPK | 1002.56348 | _ | _ |
|  | K118 | AIRNDEELNKLLGKVTIAQGGVLPNIQAVLLPKmalK | 645.36707 | _ | _ |
|  |  | VTIAQGGVLPNIQAVLLPKmalK | 725.11298 | _ | _ |
|  |  | KLLGKVTIAQGGVLPNIQAVLLPKmalKTESQK | 825.73230 | _ | _ |
|  | K119 | KVTIAQGGVLPNIQAVLLPKKmalTESQKTK | 1063.29419 | QGGVLPNIQAVLLPKKmalTESQKTKSK | 940.87396 |
|  |  | VTIAQGGVLPNIQAVLLPKKmalTESQKT | 575.92932 | VTIAQGGVLPNIQAVLLPKKmalTESQK | 920.87903 |
|  |  | VTIAQGGVLPNIQAVLLPKKmalTESQK | 690.91003 | _ | _ |
|  | K124 | KVTIAQGGVLPNIQAVLLPKKTESQKmalTK | 1063.29419 | _ | _ |
| H2b | K12 | APKKmalGSKKAITK | 1512.88628 | APKKmalGSKKAITK | 1512.88689 |
|  |  | KKmalGSKKAITK | 1348.75102 | KKmalGSKKAITK | 1348.75212 |
|  |  | _ | _ | KKmalGSKKAITK | 1362.77104 |
|  | K15 | KGSKmalKAITKAQK | 1457.86283 | PSKSAPAPKKGSKmalK | 1538.82817 |
|  |  | APKKGSKmalKAITK | 1512.88628 | KGSKmalKAITKAQK | 1457.86448 |
|  |  | KKGSKmalKAITK | 1362.77080 | APKKGSKmalKAITK | 1512.88738 |
|  |  | _ | _ | KKGSKmalKAITK | 1362.77056 |
|  | K34 | SRKmalESYSIYVYKVLKQVHPDTGISSK | 3154.72702 | SRKmalESYSIYVYKVLKQVHPDTGISSK | 3154.72080 |
|  |  | RKmalESYSIYVYK | 1549.81133 | _ | _ |
|  | K43 | _ | _ | SRKESYSIYVYKmalVLKQVHPDTGISSK | 3154.73623 |
|  | K46 | VLKmalQVHPDTGISSKAMGIMNSFVNDIFER | 3347.71694 | _ | _ |
|  | K57 | VLKQVHPDTGISSKmalAMGIMNSFVNDIFER | 3347.71694 | VLKQVHPDTGISSKmalAMGIMNSFVNDIFER | 3347.71059 |
|  | K108 | REIQTAVRLLLPGELAKmalHAVSEGTKAVTK | 3243.85488 | AKmalHAVSEGTKAVTKYTSSK | 2121.11313 |
|  |  | IQTAVRLLLPGELAKmalHAVSEGTKAVTKYTSSK | 3567.01905 | _ | _ |
|  | K116 | LPGELAKHAVSEGTKAVTKmalYTSSK | 2602.35276 | LLLPGELAKHAVSEGTKmal | 1877.06559 |
|  |  | HAVSEGTKmalAVTKYTSSK | 1907.99831 | _ | _ |
|  | K120 | HAVSEGTKAVTKmalYTSSK | 1907.99831 | _ | _ |
| H3 | K4 | TKmalQTARKSTGGK | 1474.80498 | _ | _ |
|  | K27 | _ | _ | ATKAARKmalSAPATGGVKKPHR | 2188.24888 |
|  | K37 | KSAPATGGVKKmalPHR | 1589.93630 | KSAPATGGVKKmalPHR | 1589.93569 |
|  | K56 | RYQKmalSTELLIR | 1520.86777 | _ | _ |
|  | K115 | FQSSAVMALQEASEAYLVGLFEDTNLCAIHAKmalR | 3712.83559 | FQSSAVMALQEASEAYLVGLFEDTNLCAIHAKmalR | 3726.86513 |
| H4 | K5 | RGKmalGGKGLGKGGAK | 1370.78557 | _ | _ |
|  | K8 | MSGRGKGGKmalGLGKGGAK | 1759.92851 | KGGKmalGLGKGGAK | 1373.77201 |
|  |  | KGGKmalGLGKGGAK | 1373.76972 |  |  |
|  | K12 | MSGRGKGGKGLGKmalGGAK | 1759.92851 | RGKGGKGLGKmalGGAK | 1454.84534 |
|  | K16 | GKGGKGLGKGGAKmalR | 1454.83403 | _ | _ |
|  |  | KGGKGLGKGGAKmal | 1199.71790 | _ | _ |
|  | K44 | GGVKmalRISGLIYEETRGVLKVFLENVIR | 3201.89100 | GGVKmalRISGLIYEETRGVLKVFLENVIR | 3201.88856 |
|  |  | VKmalRISGLIYEETRGVLKVFLENVIR | 3045.78962 | VKmalRISGLIYEETRGVLKVFLENVIR | 3045.78408 |
|  |  | GVKmalRISGLIYEETRGVLK | 2104.20768 | GVKmalRISGLIYEETRGVLK | 2104.20937 |
|  | K59 | ISGLIYEETRGVLKmalVFLENVIRDAVTYTEHAKR | 3948.17407 | ISGLIYEETRGVLKmalVFLENVIRDAVTYTEHAKR | 3976.21703 |
|  |  | SGLIYEETRGVLKmalVFLENVIRDAVTYTEHAK | 3721.02331 | SGLIYEETRGVLKmalVFLENVIRDAVTYTEHAK | 3721.02038 |
|  |  | VLKmalVFLENVIRDAVTYTEHAK | 2545.40371 | _ | _ |
|  | K77 | ISGLIYEETRGVLKVFLENVIRDAVTYTEHAKmalR | 3948.19790 | ISGLIYEETRGVLKVFLENVIRDAVTYTEHAKmalR | 3948.19741 |
|  |  | HAKmalRKTVTAMDVVYALKR | 2201.22947 | DAVTYTEHAKmalR | 1404.70061 |
|  |  | DAVTYTEHAKmalR | 1404.69866 | _ | _ |
|  | K79 | VTYTEHAKRKmalTVTAMDVVYALK | 2666.37961 | _ | _ |
|  |  | HAKRKmalTVTAMDVVYALKR | 2201.22617 | _ | _ |
|  |  | RKmalTVTAMDVVYALKR | 1865.05971 | _ | _ |
|  |  | KmalTVTAMDVVYALKR | 1680.91788 | _ | _ |
|  | K91 | TVTAMDVVYALKmalR | 1580.85637 | TVTAMDVVYALKmalR | 1580.85796 |

*: Protonated MW of the Primary Sequence;

#: Refers to high scoring peptide evaluated with PD software.

_: The site or peptide segment was not detected.
